# Toward a New Model of Understanding, Preventing, and Treating Adolescent Depression Focusing on Exhaustion and Stress

**DOI:** 10.3389/fpsyt.2020.00412

**Published:** 2020-05-06

**Authors:** Toon van der Gronde, Leontien Los, Arnoud Herremans, Ronald Oosting, Rafaela Zorzanelli, Toine Pieters

**Affiliations:** ^1^Freudenthal Institute and Utrecht Institute for Pharmaceutical Sciences (UIPS), Faculty of Science, Utrecht University, Utrecht, Netherlands; ^2^Department of Adolescent Psychiatry and Addiction Prevention, Brijder-Jeugd, The Hague, Netherlands; ^3^Instituto de Medicina Social, Rio de Janeiro State University, Rio de Janeiro, Brazil

**Keywords:** depression, adolescents, stress, exhaustion, treatment

## Abstract

**Objective:**

Adolescent depression is a heterogeneous disorder, with a wide variety of symptoms and inconsistent treatment response, and is not completely understood. A dysregulated stress system is a consistent finding, however, and exhaustion is a consistent trait in adolescent patients. The aim of this paper is to critically assess current hypotheses in adolescent depression research and reframe causes and treatment approaches.

**Methods:**

A mixed-method approach involved a review based on publications from PubMed, Embase and PsycInfo, and two exemplary adolescent cases.

**Results:**

Both cases show a spiral of stress and exhaustion, but with a different profile of symptoms and coping mechanisms. Reframing both cases from the perspective of coping behavior, searching for the sources of experienced stress and exhaustion, showed coping similarities. This proved essential in the successful personalized treatment and recovery process. In combination with recent evidence, both cases support the functional reframing of depression as the outcome of a stress- and exhaustion-related spiralling mechanism.

**Conclusions:**

We propose to open up a symptom-based, mood-centered view to a model in which adolescent depression is framed as a consecutive failure of stress coping mechanisms and chronic exhaustion. Addressing exhaustion and coping primarily as a treatment strategy in adolescents and young adults might work in synergy with existing treatments and improve overall outcomes. This perspective warrants further investigation.

## Introduction

Major depressive disorder (MDD) is the leading cause of disability worldwide (1), with 10 to 15% of patients proceeding to suicide (2, 3), and a substantive disease burden for adolescents and young adults (4–6). Depression is a heterogeneous group of brain disorders with varied contextualized origins, complex genetics and a neurobiology that is not completely understood. The etiology is not elucidated, and particularly for adolescents there is an evidence gap (4, 7, 8). The serendipitous discovery of first the tri- and tetracyclic antidepressants (TCAs) and later the stress-modulating serotonin reuptake inhibitors (SSRIs) led successively to the catecholamine and monoamine hypotheses of depression (9). In later years, reduced adult neurogenesis and changes in structural and functional neuronal plasticity have been linked to the onset and treatment opportunities of major depression (10, 11). Genetic research has shown that there is not a single genetic cause for depression, and all known genetic factors combined only explain a limited percentage of the variance in clinical outcomes (12, 13). The estimated heritability of depression is 35%–40%, indicating 60%–65% is explained by other factors, such as adverse life experiences (11, 14). Researchers have turned to epigenetics to develop new forms of genetic and pharmacological modeling, in an effort to describe the etiology of depression better (15). Despite many years of research by numerous investigators both in academia and industry, psychoactive targeted therapeutics with controllable and specific effects on the brain microcircuitry and chemistry did not and probably will not materialize due to the complex nature of mental disorders (16). In order to open up our thinking about MDD we take up the challenge to reframe depression, specifically focusing on adolescents.

### Symptom-Based Approach

We note that within the current framework depression is diagnosed based on the presence of a series of mood-related symptoms and their effect on daily functioning. The seven most commonly used interviews and self-report questionnaires together describe a heterogenous group of 52 symptoms, such as either high or low appetite, more or less sleep than usual, and a feeling of sadness (17). This causes differences in diagnosis based on which scale is used (18). The widely varying patterns in which these symptoms often present themselves (19, 20), and the high occurrence of several comorbidities, such as anxiety, psychosis, and autism spectrum disorder, indicate that depression is not a homogenous disease, but a continuous, heterogeneous group of disorders associated with a wide variety of different risk factors (4, 8, 21–24).

### Aim

Combined with the lack of understanding of the etiology of adolescent depression, the large variation of presentation and treatment approaches is the main driver for us to try to reframe the concept of MDD in adolescent patients. We also aim to explain why responses to treatment vary substantially and why older age is a consistent and important risk factor for a poorer MDD course (25–28). We will take a new perspective toward MDD by focusing on stress and the depressive mood related to development in adolescence. This yields a promise for novel therapeutic approaches and potential breakthroughs in depression research, treatment and prevention.

## Methods

A mixed method approach was used involving clinical investigation of adolescent case reports and a narrative review. PubMed, Embase, and PsychInfo were searched for relevant publications, with select additions of recent findings based on collective suggestions of the authors. To make sure the patient perspective is not lost when critically assessing the current framework and new possibilities, two case reports were included. Written informed consent was obtained from both subjects for the case reports.

## Depression and Stress

### Etiology

Many findings in depression research have failed the scientific test of replication. For example, the volume of the amygdala of depressed patients has been found to be increased (29) in some studies, and decreased in others (30). Patients with melancholic depression, a subtype based on symptoms, were thought to respond better to TCAs than atypical patients (hence the name) (20, 27, 31, 32), but other researchers could not replicate this finding (33–36). Plasma levels of leptin, which reduces appetite, has been found higher in melancholic patients (37), or higher in atypical patients (38). Finally, childhood trauma and/or abuse is more common in melancholic than in atypical patients (39), or *vice versa* (40).

### Stress System

One consistent finding, however, is a dysregulated stress system in depressed patients (41–44). In approximately 70% of depressed patients a dysfunction of the hypothalamic–pituitary–adrenal (HPA) axis is detected, mainly hyperactivity (38, 45). Also a disruption of the diurnal variation of cortisol is commonly seen (46, 47). Unfortunately, after decades of research efforts this finding has not resulted in a stress-targeted treatment option or a clinical test to predict treatment response (48, 49), and it remains debated whether HPA-axis dysregulation is a cause or a consequence of depression.

This does provide an important insight: depression is at least partially the result of stress and a differential dysregulation in the stress system is an important trait (38). The stressor may be in the past (e.g. childhood maltreatment or trauma) (4, 50), or acutely (e.g. dealing with new life events). The initial response of people to stress is typically a coping mechanism aimed at exerting control over the stressor either by avoiding, reducing or predicting its occurrence. Examples of such efforts are the canceling of obligations or disengagement from social interaction (51). The HPA axis exerts a fundamental role regulating both internal as external stimuli, integrating the physiopathological and behavioral dimensions of stress. We postulate that depression is the result of a failure of coping mechanisms to control the stressors and a differential dysregulation in the stress system.

### Coping Mechanisms and Exhaustion

The accumulation of stressful events, and the eventual failure of coping mechanisms to deal with the stress, can lead to exhaustion and depressive behavior. Preclinical experiments already hinted at a relation between the effectiveness of coping behavior, the effort involved and feedback on the development of gastric ulcers. Although coping efforts were effective, ulcers still developed when coping took more effort and less feedback was offered (52, 53). Preclinical evidence indicated that chronic exposure to relatively mild stressors which rats can adapt to relatively easily (e.g. tilting the cage at a slight angle, emptying a water bottle in the cage, introducing new bedding material), ultimately resulted in the development of anhedonia (50). The chronic character of having to cope with mild stressful events over and over again, and the lack of control over stressors, was sufficient for depressive symptoms to develop (54). In a forced swimming test, rats who were dosed with psilocybin developed the coping technique of floating faster than other rats, indicating a window of behavioral flexibility (55). Ketamine displayed the opposite effect, with more mobility (56). We hypothesize that depressive behavior, specifically anhedonia and withdrawal, and the consequent loss of interest and enjoyment in usual activities, is an evolutionary mechanism to guard the organism against the exhaustion that may results from excessive or chronic coping behavior. As such, depressive behavior is both an expression of psychological pressure and a physiological precaution. This substantiates the entanglement of psychological and physiological factors in MDD.

**Figure 1:**
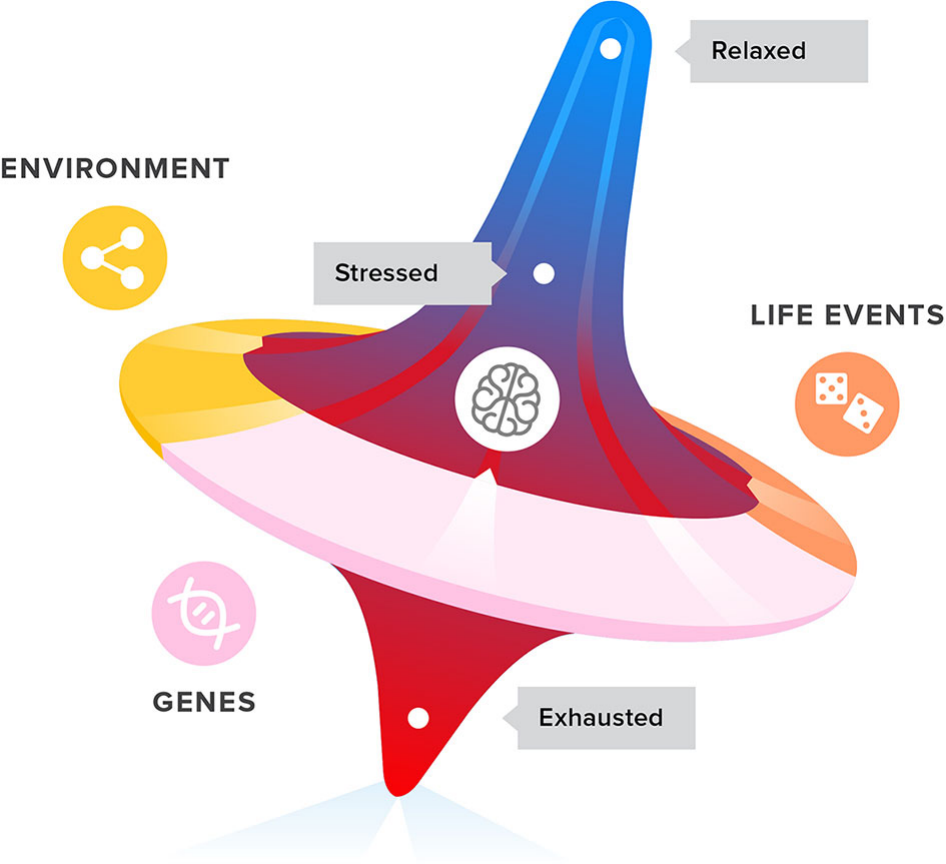
Life events, genetics, and environment all have an impact on the development of stress, coping with stress, and ultimately exhaustion and depression symptoms in adolescents and young adults.

Stress response mechanisms can change the allocation of metabolic resources in a stressful situation, where that is needed. Similarly, depression could be the expression of a forced change in allocation of attention. Depressed patients are known to ruminate, or continually analyze their problems and relive their memories (24). Anhedonia can be interpreted as a way to secure mental resources, by reducing the interest in distractions (20, 57). Depression can be seen as an exaggerated social navigating coping mechanism, caused by an accumulation of stress and a spiral of unsuccessful adaptive behaviors which leads to exhaustion. By entering a depressive mode, the organism aims to guard itself from exhaustion. The challenge is to interfere with this mood-affecting spiralling mechanism (see **Figure 1**) to prevent depression from developing. Dealing with stress and potential exhaustion, as opposed to dealing with the symptoms of depression, could prove to be an effective treatment approach.

### Treatment

There is currently only limited evidence-based rationale for choosing one treatment over another for an individual patient (31, 58–60), with no differentiated approach for adolescents or adults (6, 61). Even defining depression subtypes based on symptoms has not helped (62). Despite guidelines and evidence-based interventions, treatment is still primarily based on trial and error (63, 64), and primarily aimed at improving mood. Yet, between one third (65) to half (20, 66) of adult patients show no response to weeks of first line treatment with antidepressant drugs, and are advised to try a different antidepressant. Further, one third of all patients never reach a response after four lines of antidepressant treatment (65). The current therapeutic shortcomings are the consequences of our lack of knowledge of causes, the underlying neurobiology and chemistry (67), and risk factors that contribute to the onset and maintenance of depression. As a consequence, the treatment paradigms are oversimplified with little attention for preventive measures (68).

### Psychedelics

When Albert Hofmann, who first synthesized lysergic acid diethylamide (LSD), came in contact with it himself in 1943, he noted the hallucinogenic properties. In 1947 it was first marketed as a therapeutic drug. In the 1960s, Timothy Leary was the first to start experimenting with psilocybin combined with psychotherapy (69). In the 1950s and 1960s, LSD and psilocybin were tested in several small-scale clinical trials for anxiety, depression and addiction (70, 71). In response to increased recreational use, international legislation was introduced as part of the “war on drugs,” which brought an abrupt end to clinical research with these and similar substances in the 1970s (67, 69, 70, 72). In the last two decades, clinical trials with psychedelics have started to take place again (71). A brief overview of the indications these psychedelics have been investigated for post-2000 is indicated in **Table 1** (69, 70, 72).

**Table 1:** Overview of psychedelics and the indications they have been used for in trials since 2000.

Substance	Indication in trials
Classic psychedelics (serotonin 5-HT2A and 5-HT2C agonists)	
LSD + PT	Treatment-resistant depression, anxiety associated with life-threatening diseases
Psilocybin + PT	Treatment-resistant depression, anxiety associated with advanced-stage cancer, nicotine addiction, alcohol addiction, obsessive–compulsive disorder
Ayahuasca + PT	Treatment-resistant depression
Entactogens (mixed serotonin and dopamine reuptake inhibitors and releasers)	
MDMA + PT	Treatment-resistant depression, posttraumatic stress disorder
Dissociative anesthetics (NMDA antagonist)	
Ketamine ± PT	Treatment-resistant depression

LSD, lysergic acid diethylamide; PT, psychotherapy; MDMA, 3,4-methylenedioxymethamphetamine.

New insights in the complex etiology of depression might be offered by findings with the use of psychedelics for treatment-resistant depression (73, 74). Several psychedelics have shown to help depressive patients in a limited number of studies with small number of patients. The classic psychedelics, compounds such as LSD, psilocybin, and Ayahuasca, have diverse pharmacological profiles, including robust effects on the serotonergic system (67, 69, 72, 75). A psychedelic not acting on the serotonergic system is the dissociative anesthetic ketamine, which in subanesthetic doses acts as an antagonist on the N-methyl-D-aspartate (NMDA) receptor, a type of glutamate receptor (56, 69, 76, 77). Esketamine, the S-enantiomer of ketamine, has been approved by the Food and Drug Administration (FDA) for treatment-resistant depression (78), but ketamine has been and continues to be used off-label to treat depression too (79). This highlights that serotonergic activity, or even a mono-aminergic activity, is not required for the antidepressant effect of a psychedelic compound, further stressing the need for abandoning the old hypotheses. These hallucinogens, and the chemically related entactogen 3,4-methylenedioxy-methamphetamine (MDMA) may have a place in offering a positive experience to break the self-sustaining depressive state and allowing for introspection during psychotherapy to process stressful life-time experiences as a form of reverse medical engineering (80).

From a psychological point of view, psychedelics work through a different mechanism than classic antidepressants. Instead of the elevation of mood and the reduction of anxiety, psychedelic drugs induce a profound temporary positive experience (e.g. a mystical or religious sensation). This positive experience allows for the temporary disintegration of existing networks, which in turn facilitates reprocessing of past emotions and introspection (67). In turn, this improves the capacity to cope with stress (71). Also, the use of a psychedelic in combination with a psychotherapeutic process could have long-term effects, counteracting the effect of a negative experience and disrupting the negative and “downward spiralling” compulsive thinking (72).

### Developmental Aspects

In this article we move away from mood improvement as a primary target (81, 82). We offer an alternative integrated approach for the treatment of adolescent and young adult depression by focusing on stress factors and exhaustion reduction, seeing anhedonia and withdrawal as an evolutionary coping mechanism. This integrates approaches such as the social navigation hypothesis of Watson and Andrews (83) with cognitive bias (84) and Selye's biological stress (85, 86). With this approach we take a functional perspective, and focus on the function the depressive state provides to the adolescent patient and how it develops. This perspective is instrumental for tailor-made treatment strategies.

We will discuss these insights on the basis of two adolescent patient reports. Mood disorders have been shown to be progressive, with patients developing more complex psychopathologies over time (87, 88). Approximately 50% of patients retrospectively state that their first depressive episode occurred before the age of 20 (88, 89); another report states 50% experience that before the age of 14 (90). This further highlights the progressive nature of depression and the need for early intervention.

## Case Reports

### Case 1

A 17-year-old Caucasian woman was referred by her own general practitioner to the department of adolescent psychiatry and addiction prevention for binge drinking and daily use of marijuana. The intake together with her parents showed that the patient already had a history of moderate depression and an eating disorder, anorectic of the purging type with moderate severity. No abnormalities were reported regarding appearance, behavior, eye contact, and rapport orientation and cognition [intelligence quotient (IQ) of 127]. However, she regularly suffered from suicidal thoughts and a low ability to experience pleasure. Though she had no concrete suicide plans, in gloomy periods she showed risky behavior, like crossing a busy road without looking. She usually performed well in school, despite occasional lags in attendance, which were compensated with short periods of active study. Her mother had a history of MDD.

At the department of adolescent psychiatry and addiction prevention, we classified the addiction behavior as mild. But we also established a comorbid psychiatric and substance-use disorder profile. Thus, we chose for an integrated treatment for comorbidity that has been found to be consistently superior (91). Effective treatment for comorbid conditions combines different therapeutic modalities, i.e. psychotherapy [e.g. motivational interviewing (MI), cognitive behavioral therapy (CBT)], pharmacotherapy (e.g. antidepressants), and family therapy. Using combinations of different modalities typically increases therapeutic effect by exerting a synergistic impact on symptoms (6).

With MI, the patient was motivated to choose a first education-related treatment goal. This was to prevent school dropout at all cost. We started CBT to control her marihuana and alcohol abuse and prevent school dropout. We added medication in order to try to stabilize her mood with fluoxetine, an SSRI, which might also modulate stress. The medication initially seemed to have some effect but after two months there was a sharp mood drop, increased suicidality and aggravation of eating disorder symptoms. Eventually she had a body mass index of 16 kg/m^2^. The eating problems were mapped and analysed by an eating disorder specialist. The latter used a problem-solving approach and focused on both directive counseling and emotional support. The eating disorder specialist also advised to choose a medication with low risks of weight gain. The psychiatrist changed the medication to citalopram.

Subsequently, the treatment team focused on teaching the patient how to cope with stressful situations and the associated anxiety. The stress appeared to be mainly caused by a feeling of lack of control. The patient turned out to have a high intelligence and learning ability, but also felt that she had no control over her learning process. She had not sufficiently developed social learning strategies in her early school years. In addition, there appeared to be an issue of individuation and separation problems. These problems got worse because it was almost impossible for her parents to let her develop in her own way due to the stress they had over her suicidal thoughts, drug use, and worsening physical condition due to bad eating habits. We decided on an additional family counseling approach to address these issues.

The integrated treatment modality approach proved effective. She developed a realistic idea of what caused her stress, how she reacted situationally and improved her awareness that she tends to have control over everything. Her parents were involved in helping her developing control coping skills and checking on achievements. Because of this insight, she succeeded in maintaining her diet less strictly and experimenting with behaving differently without alcohol or drugs. Her parents saw that she was doing better and were able to release her a bit more. This increased her sense of control and provided enough space to further discover what goals she wanted to achieve. In the process her mood and her ability to experience pleasure improved significantly. She successfully passed her school exams and proceeded to university.

### Case 2

A 15-year old Caucasian girl was referred by her own general practitioner after a suicide attempt with symptoms of sadness, anxiety, and obsessive–compulsive behavior. The intake was together with her parents. She was struggling in school, despite her very supportive family. No drug abuse or other psychiatric symptoms were found. She told the counselor she tried hard, but felt that she could not keep up in school; it was never good enough, no matter how hard she tried. The counselor estimated that the school level was appropriate for the level of intelligence of the patient.

She had periods when her self-esteem was very low. During these periods she spent hours on her appearance, focusing on her hair and makeup. Her hair fell out as a result of these sessions. She could not stop herself, and always ended with self-harm. This in turn lowered her self-esteem and increased the experienced stress. She was locked in a downward spiral. Gradually her mood disorder worsened and made her passive. She no longer wanted to go to school and meet friends, but passed hours in front of the mirror. She attempted to end her life.

We hypothesized on the basis of the girl's stress complaints that she felt school, parents, and friends expected too much of her. After a neuropsychological assessment the testing showed that she had a disharmonic intelligence profile with an IQ of approximately 80 (using the Wechsler Intelligence Scale For Children-III (92)), inconsistent at all factor levels. We classified a mild intellectual developmental disorder in the conceptual and practical domain, which explained the structural struggle with the standard school curriculum instructions. We educated parents and school on how instructions might fit in better with her learning abilities and style. Her preferred method of learning new things was being shown how to do it, as opposed to having it explained to her. This led to significant stress reduction and positive school experiences. In the process her self-esteem improved, the experienced stress decreased, and her mood improved. CBT was adjusted to her learning style and was used to reduce her obsessive–compulsive behavior.

## Discussion

### Cases

Though both case reports show a different profile of symptoms and coping mechanisms, in both cases a downward spiral of stress, coping behavior, and exhaustion are central. Both patients described themselves as rarely feeling relaxed and as struggling to fulfill their daily tasks. After several years of chronic stress, a period followed in which they felt constantly exhausted.

The first patient coped with stressful situations through aberrant food intake behavior, suicidal thoughts, and mood swings. She overcompensated this restrictive behavior with recreational drug abuse. The second patient developed compulsory behavior, stress, and suicidal thoughts and overcompensated leading to self-harm. In the current framework both cases would be viewed as different and based on their symptomatology ask for different treatments. Reframing both cases, from the perspective of coping behavior, searching for the origin and sources of the experienced stress and exhaustion and coping with stressful situations, showed stress coping similarities between the two cases and proved an essential part of the personalized treatment and recovery process. Both cases support the added clinical value of the functional reframing of depression as the outcome of a mood-affecting stress and exhaustion related spiralling mechanism.

The adolescent cases presented here are good examples of how depression can be managed by relearning effective coping behavior. This prevents patients from reverting to a depressive state in order to cope with the life stressors. In more severe and chronic cases, patients suffering from difficult-to-treat or treatment resistant MDD, patients are in a deep depressive state and are not capable of learning new coping behaviors. We envision that in such situations more radical medical interventions are needed to first elevate patients from the depressive state into a state where learning new and effective coping strategies can take place. In these situations, psychedelics (e.g. ketamine) have proven to be effective to temporarily draw people out of a deep depressive state. With the support of follow-up medication and adequate psychological guidance, the patients may then develop effective coping strategies.

### Methodological Notes

As this is a narrative review, and not a formal systematic review, caution needs to be used when interpreting this data. Select literature sources have been added based on informal searches, so contradicting studies may have been missed. Similarly, the two cases were selected for their exemplary stories, not because they are typical. As such, this paper aims to provide new insights and direction for future treatments, not a definitive answer on how to improve treatment.

### Possible Implications for the Post-CoViD-19 Pandemic Period

After the Spanish flu of 1918, many infected patients developed post-viral fatigue in the following months and years. On top of that, the randomness of who was infected and who died as a result led to learned helplessness, which caused anhedonia and other depressive symptoms (93). The recent pandemic of the novel coronavirus, severe acute respiratory syndrome coronavirus 2 [SARS-CoV-2, previously known as 2019-nCov (94)], has overwhelmed healthcare facilities with the high need for acute care. This puts a large psychological toll on the entire population, with high levels of stress (95). Next to the risk of the many life events that can be expected in these situations, coping behavior—such as avoiding conflict, or searching social support—is often limited due to confinements that are put in place to prevent further spread. Furthermore, the lockdown situation and mass isolation at home in many countries may increase the risk of domestic violence and divorces. This could all lead to a rise in trauma-related stress disorders in the months and years to come. Breaking a vicious cycle of stress, inadequate coping behavior and exhaustion with a holistic view, and possibly with psychedelic-supported psychotherapy, might help treat the many psychiatric patients that can be expected.

## Conclusion

Reframing depression and shifting clinical practice to a more comprehensive and integrated look at the individual experience of a patient, including all causes for stress, pressure, and exhaustion, might be more helpful in developing promising treatment strategies. Also, treatment practices that take into account preventive mental health interventions, and that focus on stress, exhaustion, and coping strategies, could have a significant and lasting impact on many patients struggling with depression. The perspective of stress, coping, and exhaustion provides the therapist with another treatment approach that can work in synergy with the existing arsenal of therapeutic approaches, making the therapist more effective.

Increased focus is needed on support programs to help individuals develop functional coping mechanisms to deal with pressure, before more serious coping mechanisms develop in the form of withdrawal from stressful situations, compulsory behavior, or frequently occasional use of recreational drugs (96). Our intuition is that during successful treatment patients experience small successes of effective coping and re-live the rewarding properties of such experiences. Reliving experiences could repair the damaged reward mechanisms and diminishes the experienced anxiety and stress which then might subsequently drive and sustain further recovery (97). Psychedelics may offer help in breaking free from the existing cognitive bias, by facilitating introspection, re-living of past experiences, and development of new coping mechanisms.

Effective treatment strategies for adolescent and young adult depression should combine different therapeutic modalities and focus on exhaustion and sources of stress. Using a combination of treatment modalities could increase therapeutic effectiveness by improving the pace of learning new coping behaviors, exerting a synergistic impact on the developmental perspective, and breaking the downward spiral of stress and exhaustion, which eventually leads to a reduction of the depression symptoms. This might also help for other related mental disorders in adolescents and young adults where exhaustion and stress are central, such as burnout syndrome (98). But similarly, post-traumatic stress disorder, autism spectrum disorder, and generalized anxiety disorder are related to stress (24, 99–101). These disorders could also benefit from the reframing of the concept of mood and stress. We would like to offer this integrated and multidisciplinary perspective as a consideration for the development of new multimodal treatment approaches for MDD and other related psychiatric disorders.

## Data Availability Statement

All datasets generated for this study are included in the article/supplementary material.

## Ethics Statement

Written informed consent was obtained from both subjects for the case reports.

## Author Contributions

Conception or design of the work: TP, TG, LL, RO, AH, RZ. Data collection cases: LL. Data analysis and interpretation: TP, TG, LL, AH. Drafting the manuscript: TG. Critical revision of the article: TP, LL, TG, RO, AH, RZ. Final approval of the version to be published: TP, LL, TG, RO, AH, RZ

## Conflict of Interest

During the late stage development of this manuscript, TG accepted a position in oncology at AstraZeneca. AstraZeneca had no role in any aspect of this paper.

The remaining authors declare that the research was conducted in the absence of any commercial or financial relationships that could be construed as a potential conflict of interest.
